# Predictive Values of Clinical Features and Multimodal Ultrasound for Central Lymph Node Metastases in Papillary Thyroid Carcinoma

**DOI:** 10.3390/diagnostics14161770

**Published:** 2024-08-14

**Authors:** Jiarong Fu, Jinfeng Liu, Zhixiang Wang, Linxue Qian

**Affiliations:** 1 Department of Ultrasound, Beijing Friendship Hospital, Capital Medical University, Beijing 100050, China; fujrachel@163.com (J.F.); zhwang93@163.com (Z.W.); 2 Department of Interventional Ultrasound, Beijing Anzhen Hospital, Capital Medical University, Beijing 100029, China; liujinfeng1990@163.com

**Keywords:** papillary thyroid carcinoma, central lymph node metastases, multimodal ultrasound

## Abstract

Papillary thyroid carcinoma (PTC), the predominant pathological type among thyroid malignancies, is responsible for the sharp increase in thyroid cancer. Although PTC is an indolent tumor with good prognosis, 60–70% of patients still have early cervical lymph node metastasis, typically in the central compartment. Whether there is central lymph node metastasis (CLNM) or not directly affects the formulation of preoperative surgical procedures, given that such metastases have been tied to compromised overall survival and local recurrence. However, detecting CLNM before operation can be challenging due to the limited sensitivity of preoperative approaches. Prophylactic central lymph node dissection (PCLND) in the absence of clinical evidence of CLNM poses additional surgical risks. This study aims to provide a comprehensive review of the risk factors related to CLNM in PTC patients. A key focus is on utilizing multimodal ultrasound (US) for accurate prognosis of preoperative CLNM and to highlight the distinctive role of US-based characteristics for predicting CLNM.

## 1. Introduction

Thyroid cancer, the most common endocrine tumors, represents 3% of the global incidence of all malignant neoplasms. Approximately 90% of patients with thyroid cancer harbor papillary thyroid carcinoma (PTC), the most common subtype of thyroid malignancies [1]. Although most PTCs act as indolent tumors with slow progression and have a better prognosis after surgery than other tumors, the incidence of lymph node metastasis (LNM) has been reported as high as 60–70%, with malignant nodes more frequently located in the central compartment [2]. In general, LNM occurs in a stepwise fashion, which firstly occurs in the central region followed by the lateral region, but also has the condition of skip metastasis [3]. Importantly, some scholars considered the presence of central lymph node metastasis (CLNM) to be as important as primary tumors, which is associated with compromised overall survival as well as recurrence and distal metastasis [4]. Therefore, it is of great clinical importance to accurate preoperative evaluate CLNM.

The multimodal imaging assessment of the neck nodal basins has high value to detect metastatic lymph node (LN). Ultrasound (US) examination is the first-line imaging option in almost all patients, but other modalities may also be utilized. Contrast-enhanced computed tomography (CT) can provide more information and is not operator dependent, but it is more expensive and delay in radioactive iodine (RAI) therapy for patients with indications for adjuvant treatment. In comparison with qualitative CT image features, the radiomic signature of dual-energy CT iodine maps performed better in the preoperative diagnosis of cervical metastatic LN [5]. Yu et al. [6] had reported dual-energy CT reliably distinguished metastatic and non-metastatic LNs smaller than 0.5 cm in patients with PTC. Radiomics based on multiparametric magnetic resonance imaging (MRI) could also improve diagnostic efficacy of cervical LNM preoperatively [7]. Positron emitted tomography (PET) is less commonly used and generally reserved for distant metastases, while it can aid in identification of CLNM for PTC [8]. Although the imaging modalities play complementary roles in evaluating CLNM in PTCs, the preoperative diagnosis of CLNM can be still challenging. US has a high value in lateral lymph node metastasis (LLNM), with a sensitivity over 70%. However, the diagnostic accuracy of CLNM is unsatisfying, with a sensitivity below 30%, which may be due to the complicated anatomical structure of the central compartment, the shelter of bone, the strong echo of gas and the size of metastases is usually <1 cm [2]. In contrast to patients with clinically positive central neck metastasis, the advantages of prophylactic central lymph node dissection (PCLND) in clinically node-negative (cN0) patients remains a great deal of controversies. The incidence of complications after PCLND is high, such as recurrent laryngeal nerve injury, hypoparathyroidism, and chyle leakage. Owing to the unreliability of preoperative imaging examinations for operative guidance, PCLND is often considered for PTC patients throughout China and some other Asia-Pacific countries to reduce the potential possibility of hazardous re-operative surgery. At present, overdiagnosis and overtreatment of PTC are increasing rapidly worldwide and have become a major global public health challenge [9]. In recent years, thermal ablation (TA) has achieved satisfactory results in the treatment of thyroid diseases. According to the Europe 2021 Clinical Practice Guideline for the Use of Minimally Invasive Treatments in Malignant Thyroid Lesions [10], TA is highly recommended as a proper treatment in low-risk PTC patients. TA should be discouraged in PTC patients with the presence of LN or distant metastases, a cytology suspicion of aggressive subtypes, and/or imaging detection of extrathyroidal growth or multiple neoplastic foci [10]. In other words, whether there is CLNM or not directly affects the formulation of preoperative surgical procedures. Hence, it is essential to identify the PTC patients at high risk of CLNM.

High-frequency US, as the primary method, is routinely performed for differentiating or even diagnosing thyroid pathology and distinguishing tumor stages. Despite US is limited in its ability to detect CLNM, US features of primary lesions can indirectly reflect the invasiveness of tumor and predict the risk of CLNM. With the continuous progress of US technology, contrast-enhanced ultrasound (CEUS) and shear wave elastography (SWE) for PTC have immensely improved its diagnostic efficiency, suggesting that multimodal US may play great potential in predicting CLNM [11,12]. In the present work, we delve into the multimodal US diagnosis, highlighting the distinctive US-based characteristics for predicting the probability of CLNM while also summarizing the US radiomic features, which are a hotspot recently. By carefully identifying the preoperative predictive factors for CLNM, we aim to highlight the group of PTC patients with high-risk, which might help accurate prognosis of preoperative CLNM and aid in identifying approaches to individualized management.

## 2. Demographics and Clinical Characteristics

### 2.1. Age

In more recent periods, the age curve for PTC has displayed an inverse U-shape with a peak in incidence around middle age, which is likely due to the effect of intense scrutiny of the thyroid gland in middle-aged individuals [1]. Young age has been recognized as a key predictor for CLNM and the recurrence of PTC [13]. However, the age threshold varied between different studies. The eighth edition of the American Joint Committee on Cancer (AJCC) guidelines raised the age stratification to 55 years as a threshold for evaluating the clinic stage, although the age cut-off of either 45 or 55 years remains controversial for prognosis prediction [14,15,16]. Miyauchi et al. [17] found that <40 years of age as the only significant risk factor for both tumor size enlargement and novel LNM in low-risk PTC during active surveillance. They also suggested that younger age raised the risk of LNM and recurrence with a low mortality, while older age had a high recurrence rate and high mortality rate [17]. Wang et al. found the risk of CLNM was 1.5 times higher in patients aged <42 years than in those aged ≥42 years [18]. Despite the inconsistency of age cut-off value, consensus has been reached that younger age raises the risk of CLNM. Age remains as an independent indicator which should be combined with other factors to comprehensive analysis in order to thoroughly evaluate patients and make optimal clinical decisions.

### 2.2. Gender

The prevalence of thyroid cancer has an obvious gender tendency, which is over three times higher in women [19]. This is mainly because estrogen is an agonist in benign and malignant thyroid nodules. Still, men generally exhibit higher malignancy and stronger invasion than women. The risk of CLMN in males is 2.82 times that in females [20]. Even more significantly, as Tan et al. reported, the risk could be up to 4.005 times [21]. This is partly because males are more prone to have unhealthy lifestyles and high basal metabolism, which is apt to promote the proliferation of tumor cells, accelerate the spread of tumors, and increase the chance of CLNM [22]. Nixon et al. demonstrated that male sex was a risk factor for central neck node recurrence in PTC patients without PCLND and also confirmed men had a worse outcome [23]. There also exists inconsistency with aforementioned studies. Liu et al. demonstrated that there was no relation observed between gender and CLNM [24]. Sun et al. showed that female sex was an independent predictive factor of CLNM in PTC [25]. Controversies in the results might be associated with different sample types and sample sizes. However, it is undeniable that gender is a prominent patient background parameter for CLNM in PTC patients. Physical examination and imaging evaluation of cervical LN status should be emphasized preoperatively for both men and women.

### 2.3. Hashimoto’s Thyroiditis

The immunological background of Hashimoto’s thyroiditis (HT) comprises T-cell activation and a high titer of TPOAb and/or circulating TgAb [26]. The association between HT and PTC behavior has been a topic of interest in recent years, while the significance of the coexistence of HT with regard to CLNM in PTC patients continues to be debated. Some studies have suggested that HT has a protective effect on CLNM, which means that HT lymphocytic infiltrate might be related to autoimmune responses with an anticancer effect [26,27,28]. Zhou et al. [26] further demonstrated the protective role of HT in CLNM rate, which was solely related to early-stage or low-risk PTC, which might be counteracted or reversed by tumor progression or high aggressiveness. However, other studies held a different view, that CLNM was independent of HT [4,29]. Interestingly, regardless of the fact that HT was not a related risk predictor of CLNM in PTC, Liu et al. [30] suggested that PTC coexistence with HT had a greater number of LN dissections and fewer metastatic LNs in the central compartment region than those in a non-HT group. Shen et al. considered that PTC patients with HT had significantly more metastatic LNs [31]. These opposing views might be mainly due to the different selection criteria and detection methodologies adopted for HT. Additionally, tumor extent increased with each decile of preoperative thyroglobulin (TG) level enhancing. The higher the TG level, the greater the aggressiveness of the tumor. As reported previously, the serum TG level was an independent risk variable for large-number CLNMs [32]. A linear correlation between preoperative TG level, the size of the primary lesion, and the number of CLNMs was found [33]. Nevertheless, Liu et al. reported that the TG level was negatively associated with the risk of CLNM [34]. Notably, the impact of serum thyroid stimulating hormone (TSH) level on CLNM cannot be ignored. Liu et al. [35] suggested that a serum TSH level greater than 1.418 mU/L contributed to the risk of CLNM, which was similar to the study from Gao et al. [36]. Yet, other research has suggested that no significant difference in TSH level was found between CLNM and non-CLNM groups [37]. More analysis is needed to understand the associations between the serum TG or TSH level and CLNM.

### 2.4. BRAF V600E Mutation

Recently, molecular markers are increasingly being explored as a potential diagnostic and prognostic tools for PTC patients, and BRAF V600E mutation has gained wide attention in this regard as the most common alteration of PTC [38]. The scientific world seems to be divided between those that consider the BRAF V600E a reliable predictor of CLNM in PTCs and those that are skeptical on the prognostic value of this mutation. Some studies have reported the correlation between BRAF V600E mutation and CLNM, stating that PTCs with BRAF mutation presented significantly high frequencies of CLNM [39,40]. Furthermore, So et al. [41] considered that its predictive values on CLNM might be differential with different tumor sizes. However, several works have provided new evidence, in partial conflict with the previous knowledge, suggesting that BRAF does not contribute to the prediction of CLNM [42,43,44]. Gandolfi G et al. [45] further pointed out in a review that it was time to reconsider the meaning of the BRAF V600E mutation in PTC and looked for new molecular determinants that, alone or in association with BRAF V600E, might be more reliable predictors of aggressive behavior in PTCs.

## 3. Conventional Gray-Scale and Doppler US

### 3.1. Tumor Size

As always, tumor size is regarded as a valuable factor in the tumor-node-metastasis (TNM) stage, and larger tumors are prone to be more aggressive. Previous studies have confirmed the predictive value of tumor size for CLNM with no clear consensus on the cut-off point. Wu [37] and Jin [46] reported that tumor maximum diameter > 2.0 cm is an independent risk predictor of CLNM in PTC, while Zhong [47] and Wang [48] reported that tumor size of >1.0 cm is associated with CLNM in PTC. Jin et al. [46] observed that tumor size > 2.0 cm exhibited a 3.29-fold increased risk of CLNM. The risk of CLNM in PTCs with a size ≥ 1.0 cm was three times higher than those with a size < 1.0 cm, as confirmed by Wang et al. [18]. As for papillary thyroid microcarcinoma (PTMC), which refers to a PTC with a greatest diameter of 10 mm or less, most studies choose 0.5 cm as the threshold value [49,50]. There have also been other cut-off values for PTMC, such as 0.65 cm, 0.7 cm, and 0.8 cm [19,39,51]. Tumor size is closely connected to CLNM, and more clinical research is required to investigate the optimal cut-off of tumor size. Furthermore, total tumor diameter (TTD) could better assess the invasiveness of a tumor, and the risk of CLNM was 2.056 times higher in multifocal PTMC with TTD > 1 cm than in unifocal PTMC [52]. Park et al. [53] indicated that tumor volume, but not size, had predictive value with respect to CLNM. A volume > 0.385 mL was independently correlated with a higher risk of CLNM. Generally, the consensus reached is that a larger size or volume is more frequently positive for regional LNs. For larger tumors, cervical LNs should be examined carefully to enhance the detection rate of CLNM.

### 3.2. Location

**Figure 1:**
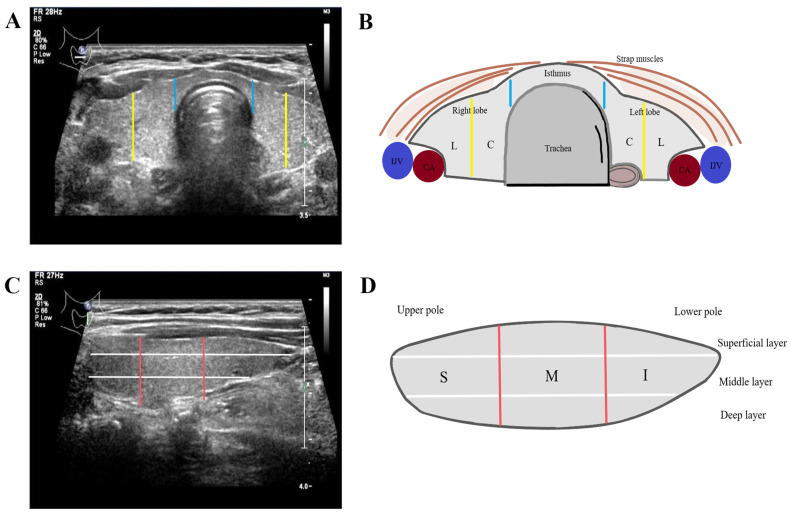
Locations of the thyroid. (**A**,**B**) In the coronal view, the thyroid gland is divided into the left lobe, right lobe, and isthmus (blue lines). Each lobe distribution is divided into lateral (L) and central (C) positions (yellow lines); (**C**,**D**) in the longitudinal view, the gland is divided into superior (S), middle (M), and inferior (I) positions (red lines); superficial layer, middle layer, deep layer (white lines). IJV, internal jugular vein; CA, carotid artery.

PTC can occur in any part of the thyroid which is composed of bilateral lobes and the isthmus (Figure 1). Controversy remains regarding the correlation between tumor site and CLNM. Yuan et al. [54] considered the location of PTC to not be correlated with CLNM, while most studies take the opposite view. PTC located in the isthmus has a low incidence, and it is prone to demonstrate more invasive characteristics compared with lobe-originating PTC. This is mainly related to its unique anatomic location, which is more likely to invade the thyroid capsule and surrounding tissues, and abundant lymphatic reflux. Previous studies have reported a higher CLNM rate in isthmic compared with lobe-originating PTC [21]. Song et al. [55] showed that the involvement of CLNM of PTC in the isthmus reached 71.1%. Zhou et al. [56] further demonstrated that isthmic PTC differs from PTC in the lobe with respect to pretracheal and bilateral paratracheal LNM, even in comparable patients. Furthermore, Sancaktar et al. [57] suggested that whether the primary tumor was in the right or left lobe had no effect on CLNM. However, Zhao et al. [58] hold a different view, that CLNM preferentially occurs from left lobe lesions, without a clear reason provided.

**Figure 2:**
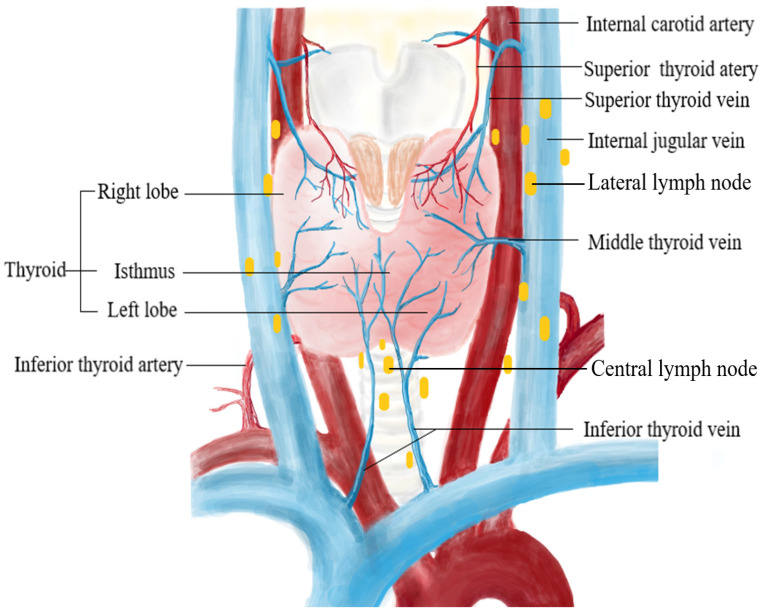
The blood supply of the thyroid gland.

The location of the primary thyroid tumor can also be denoted as upper pole, middle third, lower pole, or isthmus (Figure 1). The risk of LNM for PTC nodules at different locations might be attributed to venous reflux and different lymphatic flow pathways. Lower pole tumors are more likely transported to the central lymph nodes (CLNs) through the lymph flow along the inferior thyroid vein, while upper pole tumors are more likely transported to the lateral LNs through the lymph flow along the superior thyroid artery (Figure 2). Recent studies have proved that PTC arising in the middle or lower pole of the thyroid confers a higher risk of CLNM, while PTC located in the upper third may be a lower risk of CLNM [4,57]. A study applying 3D location suggested that middle posterior lateral (OR = 2.575), inferior anterior central (OR = 2.892), inferior posterior lateral (OR = 2.759), and isthmus tumors (OR = 4.526) have increased risk of CLNM, which means that tumors in the more interior and lower pole of the thyroid have a higher propensity to demonstrate CLNM [59]. Differently, Xiang et al. [60] reported that PTC located in the middle part of the middle third of the thyroid gland showed greater rates of CLNM than that of other locations except for isthmus. Despite this controversy existing, the location of PTC should be taken into consideration to identify occult nodal metastasis, especially when the PTC arises in the isthmus or the mid-lower part of the thyroid.

### 3.3. Multifocality and Bilaterality

Owing to the rich network of lymphatic channels in the thyroid, multifocal tumors are more invasive than unifocal tumor and prone to increase the risk of locoregional recurrence as well as LNM. Even if most tumor staging systems do not include multifocality, multifocal PTCs are more likely to be consistent with CLNM [4,39,61]. It was reported that the risk of CLNM of multifocal PTC was 2.67 times that of a single lesion [20]. However, doubt on this viewpoint was presented by Feng et al. [62], who reported no significant relation between multifocality and CLNM. Instead of being limited to discussing the difference between solitary and multifocal tumors, they further considered the significance of the number of tumor foci and found that the greater number of tumor foci a person has, the more incidence of CLNM, and the poorer prognosis will be [62]. It is not clear whether these foci represent intraglandular dissemination of a single clone or arise from distinct progenitor cells. Based on the location of lesions, multifocality can be divided into unilateral multifocality and bilateral multifocality. Zhang et al. [63] found that 84% of multifocal PTC had contralateral lobe foci. Bilateral tumors are more likely to predict the hazard of extrathyroidal invasion, CLNM, and adverse prognosis than unilateral multifocality, mainly because of their wider distribution [64]. Conversely, a meta-analysis included that bilaterality was not significantly associated with CLNM development [65]. Another meta-analysis expressed a similar view, that neither unilateral tumors nor bilateral tumors were associated with CLNM [4]. Despite the controversy, patients with multifocal or even bilateral tumors should be actively treated with a periodical follow-up.

### 3.4. Composition and Echogenicity

Most thyroid carcinomas manifest as solid nodule. Previous studies have indicated that solid components have not been proven to be associated with CLNM [66]. Conversely, Tao et al. [67] reported that solid composition was an independent factor for CLNM, which might relate to the density of blood flow. Another study considered that the cystic change of thyroid nodules raises the risk of CLNM [68]. The exact correlation between the composition of PTCs and CLNM has not been completely identified to date.

Echogenicity is usually assigned by using the adjacent thyroid parenchyma as a frame of reference. Marked hypoechogenicity is defined as decreased echogenicity when compared with the surrounding strap muscle. Wang et al. [18] indicated that there was no correlation between echogenicity and CLNM, although hypoechoic or very hypoechoic conditions were independently associated with the diagnosis of PTC. Kim et al. [69] suggested texture analysis based on US imaging was not useful for predicting CLNM in PTC. Yet, marked hypoechogenicity as an independent predictor for CLNM was also reported in previous research [39,70]. Other studies reported that patients with US images showing a complex echo pattern rather than homogeneity are more likely to have LN metastasis [71].

### 3.5. Margin

**Figure 3:**
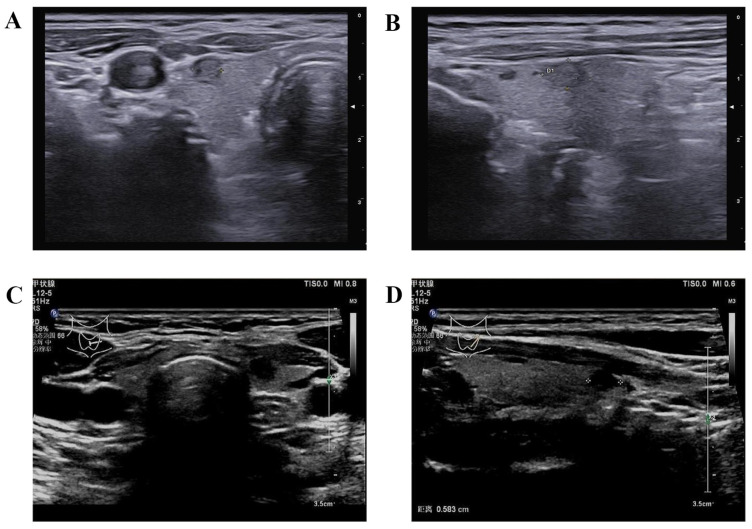
Capsular contact and capsular abutment. (**A**,**B**) Capsular abutment is defined as the lack of intervening thyroid tissue between the thyroid tumor and thyroid capsule; (**C**,**D**) capsular disruption is defined as the loss of the perithyroidal hyperechogenic line at site of contact with thyroid tumor. “甲状腺”: Thyroid; “动态范围”: Dynamic range (Dym R); “余辉 中”: Persistence Medium (P Med); “分辨率”: Resolution (Res); “距离”: Dist.

Margin is commonly used to analyze the invasiveness of the tumor. Thyroid nodules with a smooth margin generally indicate slow growth and low invasiveness, while high invasiveness is represented by a lobulated or irregular margin which warrants two points on the American College of Radiology Thyroid Image Reporting and Data Systems (ACR TI-RADS) scale [70,72]. Feng et al. [62] elucidated that a lobulated or irregular margin detected by US was an independent risk factor of CLNM (OR = 1.704, 95% CI: 1.205–2.410, *p* = 0.003). The invasive growth characteristics of the tumor could also cause the loss of a clear margin between the lesion and the surrounding tissues. Multivariate analysis had suggested that an ill-defined margin is an independent risk factor of CLNM, although it receives zero points for a margin as a non-discriminatory feature in TI-RADS [72,73]. Extrathyroidal extension (ETE), a three-point feature in TI-RADS, is an involvement of perithyroidal structures by direct extension from the primary thyroid tumor. The presence of ETE is pathognomonic for malignancy, and the eighth edition of the AJCC staging system defined minor ETE and gross ETE separately as staging variables [14]. Currently, ETE is widely regarded as an independent predictor of CLNM [4,37]. ETE on a microscope can be reliably evaluated by preoperative US, which is valuable for further clinical treatment [74]. Shorter, closer proximity to the thyroid capsule results in greater risk of CLNM. Seong et al. [75] suggested that occult CLNM was associated with a distance from the capsule < 1.9 mm. This is mainly due to the fact that a peripheral tumor location might allow PTC cells to acquire aggressive characteristics, resulting in ETE and CLNM. The presence of capsular contact and capsular abutment can provide useful predictive information about ETE (Figure 3). Cai et al. [76] found that contact of >25% with the adjacent capsule was an independent predictor for CLNM. Moreover, Feng et al. [77] revealed that ETE and capsular contact >50% were independent risk factors for high-volume LNM. Wu et al. [37] used the distance ratio pattern to show that the contact of nodules with the thyroid capsule and the extracapsular spread of the nodules were significantly associated with CLNM. Furthermore, the number of tumor-contacting surfaces, as a new observation indicator, has been proven to be an independent predictive factor of CLNM [74]. Generally, the margin of PTC is a strong indicator for the presence of CLNM, especially ETE.

### 3.6. Shape

An aspect ratio ≥ 1 is a highly specific index in identifying malignant thyroid nodules. A nodule that is grown more front-to-back than side-to-side suggests that it has violated tissue planes and is therefore suspicious. In a recent study of 645 patients with PTC, aspect ratio was an independent risk variable for large-number CLNMs in univariate and multivariate analysis [32]. Similarly, Zhou et al. [70] retrospectively reviewed 2376 patients with PTC and found that aspect ratio was independently associated with CLNM metastatic status. They further reported that the cross-sectional aspect ratio compared with the longitudinal aspect had a more effective predictive value for CLNM with larger thyroid tumors (PTC above pT1a). Conversely, Liu et al. [78] reported that no statistical significance was seen in 1657 PTC patients with an aspect ratio ≥ 1 in predicting CLNM. Notably, Luo et al. [79] revealed that PTCs located in the isthmus more frequently had the wider-than-tall shape when they had LNM and tumor capsular invasion. The characteristics of wider-than-tall shape seem to be due to narrow space in the isthmus, which limits the longitudinal growth of PTC, while it does not hinder transverse growth. The larger the isthmus PTC, the more likely the wider-than-tall shape. In terms of the regularity of shape, Zhong et al. [47] reported that irregular shape can not only be used to estimate the nature of thyroid nodules but also help to make a preoperative judgment about CLNM in patients with PTC, while Cai et al. [76] held the opposite view that there was no significant difference in nodule shape (irregular vs. regular) between positive and negative groups. Shape is known as an important predictor for patients with PTC, but its predictive value in CLNM awaits further study.

### 3.7. Calcification

**Figure 4:**
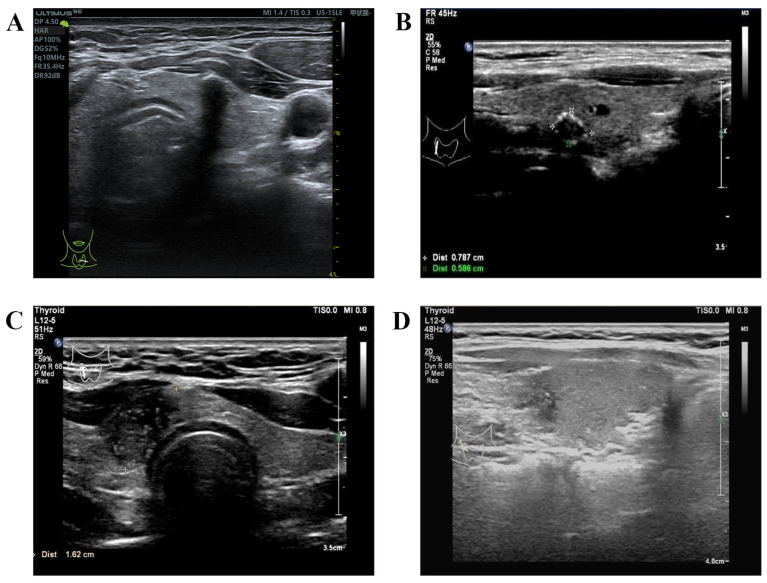
The different types of calcifications. (**A**) Macrocalcifications; (**B**) peripheral (rim) calcifications; (**C**) punctate echogenic foci; (**D**) thyroid parenchyma microcalcification. “甲状腺”: Thyroid.

Calcification is a helpful criterion in the discrimination of malignant from benign thyroid nodules, which generally manifests as echogenic foci on US. Microcalcification usually corresponds to thyroid psammoma bodies (PBs), which are suggestive of highly invasive PTC. The mechanism of microcalcification formation in PTC is thought to be formed by vascular thrombosis, calcification, and tumor cell necrosis or necrosis and calcification in intra-lymphatic tumor thrombi [80,81]. American Thyroid Association guidelines (ATA-2015) categorized microcalcifications and rim calcifications with small extrusive soft tissue components as highly suspicious signs [82]. According to the guidance of the ACR TI-RADS, echogenic foci had been divided into four categories and assigned different values, respectively: no or large comet-tail artifacts, zero points; macrocalcifications, one point; peripheral (rim) calcifications, two points; and punctate echogenic foci, three points [72] (Figure 4). A growing number of studies have believed that microcalcification is a significant independent predictor for CLNM [37,47,83]. Wang et al. [18] studied 950 consecutive patients and concluded that PTCs with focal, diffuse punctate echogenic foci, or mixed echogenic foci, were associated with a higher risk of CLNM. Furthermore, the greater the number and distribution of punctate echogenic foci, the higher the possibility of CLNM. A multivariate analysis revealed that CLNM was positively correlated with the number of microcalcifications greater than or equal to five [84]. Ha et al. [85] reported that microcalcification and mixed calcification types of PTC showed more aggressive phenotypes and more advanced TNM stage than those with no calcification and macrocalcification. Interestingly, Wang et al. [86] suggested that more attention should be paid to calcifications showing irregular and long trip shapes or central locations, which are associated with the risk of CLNM in PTC patients. Furthermore, intratumoral microcalcification and thyroid parenchyma microcalcification have been confirmed as independent predictors of CLNM [80] (Figure 4). Therefore, US can better predict the risk of CLNM in PTC to a certain extent using different types of calcifications.

### 3.8. LN Status on US

Sonography is the preferred screening modality for the preoperative evaluation of LN status, though with limited sensitivity [87]. The US features of round shape, cystic change, calcification, loss of echogenic fatty hilum, and abnormal vascularity are useful sonographic criteria for the diagnosis of suspected LNs [88]. It has been widely recognized that abnormal LNs on preoperative US are the strongest independent predictor for postoperatively identified CLNM [89,90]. Moreover, Gao et al. [91] prospectively found that the number of CLNs on preoperative US could provide additional information that the criteria of ≥2 and ≥3 CLNs might serve as ancillary preoperative markers for predicting CLNM and large-volume CLNM in PTCs. Furthermore, some scholars have also used multimodal US to improve the diagnostic accuracy of abnormal LNs [92,93]. In short, a preoperative US examination is essential for the evaluation of LN status, which is of great significance for the selection of surgical methods.

### 3.9. Doppler US

Angiogenesis is a precursor for regional LNM and reflects microvessel density in local tumor progression. The abundance of blood flow signals often means the enhancement of tumor cell contact in peripheral areas with the lymphatic tract or an increment of lymph–vessel–venous connections, which might result in more intravascularly metastasized cells entering the lymphatic system, that is, there is more possibility of CLNM [94]. With Doppler US, tumoral vascularity can be calculated preoperatively. As some researchers recently noted, the angiogenesis characteristics of PTC were closely associated with CLNM that was more common in patients with active angiogenesis [83,95,96], while some scholars held the opposite views; they thought that the internal tumor blood supply might be vital only in PTC, but might not be associated with lymphatic system metastasis [97,98]. Furthermore, Xia et al. [99] revealed that vascularity and blood supply in US were positively correlated with tumor size to some extent, which was rare in smaller tumors due to difficulty in low-velocity blood signal presenting. As for the blood flow resistance index (RI) of tumors, Zhan et al. [100] reported that a higher RI was more common in a metastatic group, even if there was no significant difference in the resistive index, and they proposed that this might due to the spatial heterogeneity of the microcirculation in a tumor. Yet, Liu et al. [98] found that RI could be used as an independent risk factor for CLNM, which might be related to the oppression of small blood vessels. Guang et al. [101] used the superb microvascular imaging (SMI) technique to evaluate microvessel information in PTC nodules and indicated that the characteristics of SMI (Grade II) were independent predictors for CLNM, suggesting that the degree of rich and disorderly blood flow in PTCs could be invaluable to predict CLNM. Similar conclusions were drawn by Wang et al. [48], who applied the Angio PLUS (AP) microvascular Doppler ultrasound technique, showing that AP vascularization in PTCs differed significantly between patients with and without CLNM. Conversely, Shin [102] and Lee [103] used a new quantitative US parameter, the vascular index (VI), to measure tumoral vascularity more objectively and found that the VI was not significantly associated with CLNM. The possible causes included differences in sample selection, the limitations in representing vascularity, and the important role of lymphangiogenesis in the dissemination of PTC. Even without unified opinions formed, intranodular vascularity should be careful considered in preoperative evaluation for possible CLNM.

## 4. Multimodal US

### 4.1. CEUS

CEUS, a pure blood pool imaging technique, can evaluate thyroid tumors qualitatively and quantitatively by providing visualization of the macro- and micro-vascularization of the tumor [104]. In recent years, CEUS has been developed rapidly to predict the aggressiveness of PTCs [105]. In terms of enhancement intensity, more research has believed that hyper- or iso-enhancement at peak time could be an independent risk factor for CLNM, indicating that the invasion and metastasis of cervical LNs might rely on angiogenesis of PTC lesions [11,106]. Zhan et al. [107] further reported that a higher peak intensity indicates a higher possibility of CLNM. Yet, some studies hold the opposite view that the enhancement pattern of CEUS is not related to CLNM, which is probably due to the different data sets and subjective judgment [67,108]. Still, Tao et al. [67] found that PTC with a peak of the nodule interior of 28.38 or greater on CEUS was reliably prone to CLNM. Furthermore, the relations between tumor size and the degree of enhancement need to be taken into consideration. Zhang et al. [109] reported that internal low-enhancement patterns were a valuable predictor for CLNM, mainly due to PTC with a diameter less than 20 mm accounting for an overwhelming proportion. Low-enhancement was apt to occur in microcarcinomas (<10 mm diameter) with small and immature vascular networks, and iso-enhancement or high-enhancement was commonly observed in large PTC (10–20 mm or >20 mm diameter), rich, and complicated vascular network.

As for the degree of homogeneity, Wang et al. [110] believed that homogeneous enhancement was more frequently found in patients with CLNM. Zhang et al. [109] hold a different view, that PTCs with an internal heterogeneous enhancement pattern were predictive for the presence of CLNM. As PTC develops further, malignancy infiltration causes neovascular damage, and perfusion defects within lesions were typically manifested as heterogeneous enhancement patterns. However, others have thought that homogeneity in CEUS had no significant difference between metastasis and non-metastasis groups [48]. 

In other features on CEUS, no agreement has yet been reached. Zhang et al. [109] suggested that an irregular no-enhancement ring may predict the risk of CLNM, which might be due to the invasion of primary lesions leading to the interstitial fibrosis and hyaline necrosis occurring in the adjacent tissue. Liu et al. [111] reported that higher and faster enhancements were more common during the early ascending period in a metastasis group. In detailed parameter estimation, a study stated that a higher rise time raised the risk of CLNM, while time to peak, mean transit time, velocity of intensity increase, and velocity of intensity decrease were not associated with CLNM [112]. Furthermore, Wang et al. [48] found that there was no statistical difference in contrast agent arrival time, enhancement direction, and ring enhancement at CEUS between non-metastasis and metastasis groups. In addition, there was increasing evidence focused on the prediction of ETE at CEUS for CLNM, which showed more diagnostic value than conventional US [48,101].

Due to the subjectivity of observation, the results of judging the qualitative indicators on CEUS may show inconformity across different studies. Notwithstanding, it is undeniable that CEUS, as a useful tool for the prognosis of PTC, might be valuable for formulating appropriate strategies and avoiding second central compartment node dissection to some degree.

### 4.2. SWE

Stiffness is a key property of abnormal tissues and organs. Elastography has emerged as a complementary tool for grayscale US in distinguishing benign from malignant thyroid nodules [113,114]. As recent studies have highlighted, SWE is a new elastic imaging technology which can quantitatively measure tissue hardness with adequate repeatability [115]. Some studies have believed that SWE can be used to predict CLNM in PTC patients. Park et al. [116] found that CLNM was related to Emean and Emax, and LLNM was related to Emin. The predictive value of SWE parameters has also been confirmed by Li et al. [115], suggesting that the Emax, Emean, and Emin indices were correlated with CLNM, especially Emax > 59.0 kPa, which was associated with a 4.93-fold increased risk of CLNM. Wan et al. [84] reported that a larger shear wave velocity (SWV) mean and SWV ratio are associated with CLNM. Zhong et al. [117] suggested that an SWV ratio > 1.3 predicted CLNM risk in patients with PTC. That is, the harder the tumor is, the higher risk that CLNM occurs. Presumably, the invasive tumor leads to complex matrix reactions that remodel collagen and increase hardness. In contrast, others have contended that SWE elasticity parameters are not independent predictive factors of CLNM [118,119,120]. One possible explanation is that SWE parameters are relatively vulnerable to many factors such as tumor size, tumor depth, pre-compression, neck morphology, and fibrosis from previous neck surgery. Moreover, the higher the T stage, the greater the degree of tumor necrosis, and thus the lower the elasticity value. Based on strain ultrasound elastography (SUE), strain rate ratio (SRR) and elasticity score were also helpful in predicting CLNM [74,118]. Moon et al. [121] showed that a hard malignancy on the Rago score of elastography was an independent factor for predicting ETE on pathology, rather than CLNM. Xu et al. [122], using acoustic radiation force impulse (ARFI) elastography, found that a virtual touch tissue imaging area ratio (VAR) > 1 was a risk factor for CLNM and a hard malignancy on elastography scores was not associated with CLNM. The relationship between thyroid cancer stiffness and the likelihood of CLNM remains to be elucidated.

## 5. US Radiomics

US radiomics as a novel approach refers to the high-throughput mining of quantitative image features from medical images, translating those unseen aspects of the images to a readable value by clinicians [123]. Machine learning based on radiomics is continuing to gain ground in the medical field, allowing improvements in predictive, diagnostic, and prognostic accuracy [124]. At present, many studies have borne out that US radiomics of PTC has the potential to predict CLNM. Agyekum et al. [123] developed a CLNM prediction model based on clinical-US radiomic features with an AUC of 0.71. Furthermore, Liu et al. [125] and Xue et al. [126] attempted to use a multimodality US-based radiomic model, achieving a better metastasis estimation performance than the model based on B-US alone. There is also research based on deep learning. Deep learning algorithms have obvious benefits compared with traditional machine learning approaches in that they reduce the need for domain expertise and the extraction of hardcore features [123]. Wang et al. [127] built a deep learning-based multifeatured integration prediction model to predict CLNM in PTCs which achieved AUCs of 0.89 in the training set and 0.78 in the test set, which was declared a high prediction efficacy for CLNM.

## 6. Conclusions

With TA guided by US or active surveillance replacing surgery for PTCs, accurate prediction of CLNM during diagnosis appears particularly important. Age, tumor size, tumor location (isthmus or the mid-lower part of the thyroid), ETE, calcification, and LN status effects on US should be fully considered to predict CLNM in PTC patients. The relationship between multi-modal US features and CLNM remains to be further elucidated. The combination of different factors in comprehensive analysis is required to preoperatively assess whether a PTC patient is at high risk of CLNM and assist clinicians in decision making.

## Data Availability

Not applicable.

## Abbreviations

PTC	papillary thyroid carcinoma
LNM	lymph node metastasis
CLNM	central lymph node metastasis
LN	lymph node
US	ultrasound
CT	computed tomography
RAI	radioactive iodine
MRI	magnetic resonance imaging
PET	positron emitted tomography
LLNM	lateral lymph node metastasis
PCLND	prophylactic central lymph node dissection
cN0	clinically node-negative
TA	thermal ablation
CEUS	contrast-enhanced ultrasound
SWE	shear wave elastography
AJCC	The American Joint Committee on Cancer
HT	Hashimoto’s thyroiditis
TG	thyroglobulin
TSH	thyroid stimulating hormone
TNM	tumor-node-metastasis
PTMC	papillary thyroid microcarcinoma
TTD	total tumor diameter
CLNs	central lymph nodes
ACR TI-RADS	The American College of Radiology Thyroid Image Reporting and Data Systems
ETE	extrathyroidal extension
PBs	psammoma bodies
ATA	the American Thyroid Association guidelines
RI	resistance index
SMI	superb microvascular imaging
AP	Angio PLUS
VI	vascular index
SWV	shear wave velocity
SUE	strain ultrasound elastography
SRR	strain rate ratio
ARFI	acoustic radiation force impulse
VAR	virtual touch tissue imaging area ratio
